# Curvature reduces bending strains in the quokka femur

**DOI:** 10.7717/peerj.3100

**Published:** 2017-03-22

**Authors:** Kyle McCabe, Keith Henderson, Jess Pantinople, Hazel L. Richards, Nick Milne

**Affiliations:** School of Anatomy, Physiology and Human Biology, University of Western Australia, Perth, Western Australia, Australia

**Keywords:** Femur, Finite elements analysis, Curved bones, Strain reduction

## Abstract

This study explores how curvature in the quokka femur may help to reduce bending strain during locomotion. The quokka is a small wallaby, but the curvature of the femur and the muscles active during stance phase are similar to most quadrupedal mammals. Our hypothesis is that the action of hip extensor and ankle plantarflexor muscles during stance phase place cranial bending strains that act to reduce the caudal curvature of the femur. Knee extensors and biarticular muscles that span the femur longitudinally create caudal bending strains in the caudally curved (concave caudal side) bone. These opposing strains can balance each other and result in less strain on the bone. We test this idea by comparing the performance of a normally curved finite element model of the quokka femur to a digitally straightened version of the same bone. The normally curved model is indeed less strained than the straightened version. To further examine the relationship between curvature and the strains in the femoral models, we also tested an extra-curved and a reverse-curved version with the same loads. There appears to be a linear relationship between the curvature and the strains experienced by the models. These results demonstrate that longitudinal curvature in bones may be a manipulable mechanism whereby bone can induce a strain gradient to oppose strains induced by habitual loading.

## Introduction

Many long bones in animal limbs are curved, but such curvature is thought to reduce the bones strength under longitudinal loading. Several authors have constructed hypotheses to explain this apparent paradox of bone curvature. It has been suggested that curvature may serve to induce strain (Lanyon, 1980), accommodate musculature (Lanyon, 1980), or warn of impending strain limits (Currey, 1984). The most widely accepted hypothesis suggests that bone curvature is the result of a trade-off between strength and predictable bending (Bertram & Biewener, 1988). A curved bone, while not as strong as a straight bone, will only ever experience unidirectional bending strain. In contrast, straight bones are liable to experience bending in any direction. While the “predictability hypothesis” is potentially powerful, it lacks an operational mechanism.

Frost (1964) and Pauwels (1980) separately suggested that the combination of muscle bending and bending due to longitudinal loading of bone curvature serves to moderate the net strain experienced by bone. Pauwels (1980) demonstrated that bending moments of equal magnitude but opposite orientation will cancel out, and Frost (1964) proposed a cellular mechanism whereby bones may develop curvature as a result of habitual loading. Both authors illustrated their arguments using thought experiments; however, their ideas remained experimentally unvalidated until recently.

Milne (2016), drawing on the ideas of Frost (1964) and Pauwels (1980), recently suggested that curvature in long bones is a strain-reducing adaptation to habitual loading. A llama (*Lama guanicoe*) radioulna was used to illustrate this hypothesis. In terrestrial quadrupeds, the action of triceps pulling on the olecranon process exerts a substantial bending load upon the radioulna simply to maintain stance. This habitual loading induces a cranial-bending strain gradient in the bone (tending to make it cranially concave). Milne used finite element analysis to demonstrate that the normally curved llama radioulna is subject to less bending strain than a straightened version of the same bone when subject to loading from triceps and longitudinal forces. He argued that this because the radioulna in these species—indeed in most terrestrial quadrupeds—is caudally curved (with a caudal-facing concavity). When this curvature is subject to longitudinal loading from gravity, joint reaction forces or other muscular forces, it deforms predictably—it will be subject to caudal bending. The cranial bending due to triceps and the curvature-induced caudal bending are in direct opposition, and so will cancel, leaving the bone in a neutral or reduced bending state.

There have been a number of strain gauge studies examining the radius and tibia of quadrupedal animals during locomotion (Lanyon & Baggott, 1976; Lanyon & Bourn, 1979; Lanyon, Magee & Baggott, 1979; Biewener et al., 1983; Swartz, Bertram & Biewener, 1989). Recently Copploe and colleagues (2015) placed strain gauges on the femur of and armadillo and found predominantly mediolateral bending in the medially (concave) curved armadillo femur. Jade and colleagues (2014) used finite elements analysis to examine Bertram & Biewener’s (1988) predictability hypothesis and showed that the caudally curved human femur is placed in caudal bending when subjected to longitudinal loading.

When a terrestrial animal bears weight on its hindlimb, the hip and knee must resist flexion and the ankle must resist dorsiflexion. The active muscles are the hip extensors (hamstrings, quadratus femoris and the adductors), the knee extensors (quadriceps) and the calf muscles joining the Achilles tendon (Elftman, 1929; Freedman & Twomey, 1979). At the beginning of stance these muscles are expected to act eccentrically as the limb absorbs ground reaction forces. These muscles continue to act, and work concentrically towards the end of stance. The muscles that attach to the posterior aspect of the femur (quadratus femoris and adductors superiorly, gastrocnemius and plantaris inferiorly) are expected to cause cranial bending (compression on the cranial side). In the caudally-curved femur, the longitudinal forces, hamstrings, and quadriceps are expected to induce caudal bending forces, thus balancing this cranial bending. However, if the femur were not caudally curved, these longitudinal forces would be less able to counter cranial bending.

This study used finite element analysis to compare the strains in models of the femur of a quokka (*Setonix brachyurus*) with varying degrees of curvature. A finite element model of a normal femur was warped to produce straight, extra-curved and reverse-curved models of the original femur. The straight model provided an immediate comparison to the normal model. This comparison was used to address the question: would a curved bone be less strained than an equivalent straight bone? It is expected that both models would be subject to similar degrees of cranial bending due to the hip extensors and ankle plantarflexors. The normal (caudally curved) model is also expected to generate a caudal bending moment (the curved bone effect) in opposition to the cranial bending. The straight model, which cannot generate significant caudal bending, would be subject only to cranial bending, and so would be more strained than the normal model. The two other form variants were descriptive of different degrees of sagittal curvature, and were included to determine if there is a relationship between the magnitude of the curvature and the strength of the curved bone effect.

## Materials and Methods

**Figure 1:**
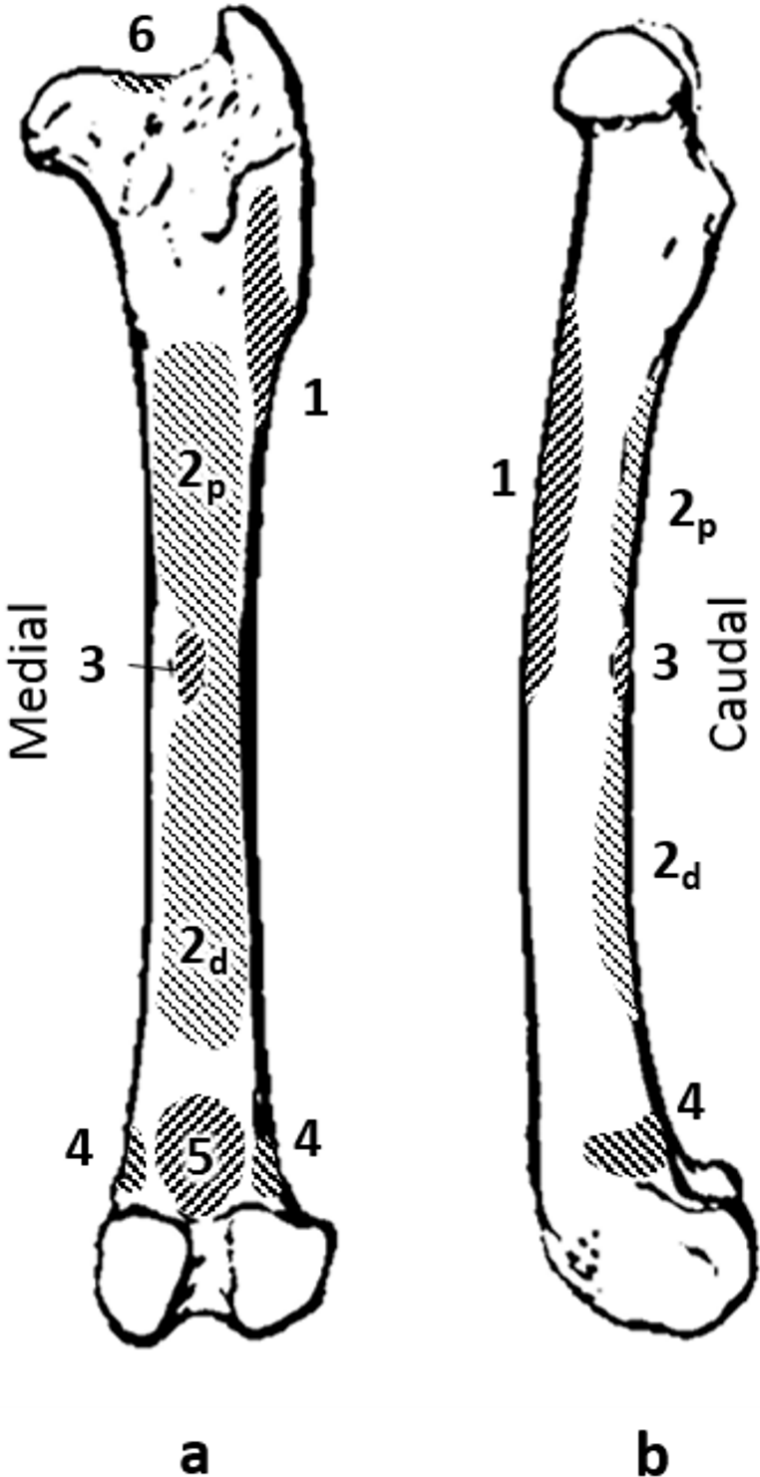
Quokka femur with muscle attachments. (A) caudal view. (B) medial view. Muscles: 1, vasti; 2p, proximal adductor; 2d, distal adductor; 3, quadratus femoris; 4, gastrocnemius; 5, plantaris; 6, site of longitudinal load.

We dissected a formalin-fixed adult female quokka hindlimb to identify the main muscles that act on the femur. For each muscle the area of attachment to the femur was recorded using a microscribe G2X (Solution Technologies Inc., Sun Prairie, WI, USA) (Fig. 1.). The microscribe was also used to record the direction of muscle action in order to construct the force vectors used in the finite element (FE) models. Each muscle was removed and the mass, volume, fibre length and pennation angle were recorded. Fascicle length and pennation angle were calculated using coordinate data taken with the Microscribe. These data were used to calculate the physiological cross sectional area (PCSA), which is proportional to the maximum force the muscle can produce (Sacks & Roy, 1982; Powell et al., 1984; Anapol & Barry, 1996; Fukunaga et al., 1996; McGowan, Skinner & Biewener, 2008).

Physiological cross sectional area (cm^2^) was calculated according to the accepted formula, where *m* is muscle mass (g); *θ*, pennation angle (°); *ρ*, muscle density (g. cm-3); and *l*, fascicle length (cm) (Sacks & Roy, 1982; Anapol & Barry, 1996; Fukunaga et al., 1996; McGowan, Skinner & Biewener, 2008). Muscle density was assumed to be 1.056 g.cm-3 (Powell et al., 1984). Maximal tetanic tension, denoted ‘*F*_*max*_’ (N), was calculated as the product of PCSA and specific force (*F*_*s*_ = 22.5 N cm-2) (Powell et al., 1984).

\begin{eqnarray*}PCSA& = \frac{m.\cos \nolimits \theta }{\rho .l} \end{eqnarray*}

\begin{eqnarray*}{F}_{max}& ={F}_{s}.PCSA. \end{eqnarray*}

A computed tomography (CT) scan of a dry quokka femur was used to create a finite element model. The scan resolution was 0.3 mm and the slice thickness was 0.33 mm. The CT stack was segmented in Amira 3.1 (Mercury Computer Systems Inc., Andover, MA, USA). The cancellous ends of bone were treated as solid but the medullary cavity in the shaft was retained. Amira was also used to place 83 landmarks evenly over the surface; these landmarks were used in the geometric morphometric (GM) analysis of deformation in the FE analyses (FEAs). The three-dimensional volume data were exported as a bitmap stack and then converted into an eight-noded (cubic) finite element mesh. The mesh was assigned homogenous isotropic material properties commonly attributed to bone (Young’s modulus 17 GPa and Poisson’s ratio of 0.3) (O’Higgins & Milne, 2013). The linear FEA was conducted using vox-FE, a non-commercial FEA software (Fagan et al., 2007).

The models were constrained at nodes on the distal aspect of the femoral condyles, and a sliding constraint was placed on the medial side of the femoral head that only allowed the head to slide towards the fixed constraint at the knee. Initially, a number of models with different voxel sizes (0.2 0.3, 0.4, 0.5, 0.6 and 0.7 mm) were created and tested by applying a longitudinal force through the femoral head, directed towards the constraints at the knee. The 0.2 and 0.3 mm models gave similar results. The 0.4 and 0.5 mm models gave slightly lower strain values, indicating increased stiffness, and the 0.6 and 0.7 mm models were increasingly stiff. Accordingly, the 0.3 mm model was used as it gave similar results to the 0.2 mm model, but with reduced computational time (the 0.3 mm models contained about 300,000 elements).

To make models of the quokka femur with different curvatures, a caudally directed load was applied to the caudal aspect of the model. After running the analysis in vox-FE, the original and new values for the 83 landmarks were analysed in the EVAN tool box (http://www.evan-society.org) together with a Stanford ply surface file representing the femoral model. The deformation along the first principal component (PC) could then be exaggerated and the resulting femoral shape examined. In this way, coordinates representing a straight version of the quokka femur were extracted. Those new coordinates were used in Amira to warp a surface file of the original model to the straightened state. The “scan convert surface” module was used to produce an Amira mesh file which was in turn was converted to a new finite element mesh model. Extra-curved and reverse-curved models were created in the same way, by doubling the deformation in the case of the reverse-curved, and reversing the deformation on the normal model in the case of the extra-curved model. The curvature of the resulting models, as well as a sample of eight actual quokka femora, was measured according to methods adapted from Richmond & Whalen (2001). A grid of 11 evenly-spaced parallel lines was superimposed over a photo of the lateral aspect of the models. The lines were perpendicular to the long axis of the bone, and were positioned such that the proximal line sat at the distal margin of the greater trochanter, and the distal line sat on the distal epiphyseal line. The intersections of these lines with the anterior margin of the model were digitised using tpsDig 2 (Rohlf, 2015). The coordinates of these points were translated, rotated and scaled such that the most distal landmark sat at (0,0), and the most proximal landmark sat at (1,0) on a Cartesian plane. The largest absolute *y* value was used as the index of the bone’s curvature.

Forces representing the adductors and quadratus femoris (the adductor group), gastrocnemius and plantaris (gastrocnemius group), the vasti, and longitudinal forces were applied individually and additively. In pilot analyses we used the maximum muscle forces as estimated by PCSA but these produced gross deformations of FE models that were unrealistic and made the results unreliable. The magnitude of the forces used was 10% of the maximum as calculated from the PCSA. The longitudinal force was calculated as 10% of the sum forces of the biarticular muscles that cross the femur (rectus femoris and the hamstrings). This force was applied at the lateral edge of the proximal articular surface and directed between the condyle constraints distally (Fig. 1.).

The results are presented in the form of compressive and tensile strain maps, and also by GM analysis of the deformation based on the 83 landmark coordinates after each loading state.

## Results

The data from the muscle dissections together with the calculated PCSA and estimated maximal forces of each muscle are provided in Table 1.

**Table 1:** Measurements from dissected specimen and PCSA and force calculations. Measurements of mass, volume, pennation angle and fibre length taken from the dissected specimen. Measurements are taken from large muscles attaching to the femur, as well as the large muscles that span the hip and knee. The table lists the PCSA that was calculated for each muscle, as well as the maximal force each muscle is capable of producing. The muscles below the line are biarticular muscles used to estimate the longitudinal force.

Muscle	Angle (°)	Length (cm)	Volume (mL)	Mass (g)	PCSA (cm^2^)	Max Force (N)
*m. vasti*	20	2.3	20.0	21.1	8.25	185.70
*m. quadratus femoris*	18	3.5	7.0	7.5	1.94	43.71
*m. adductor*						
distal part	0	8.9	17.0	18.2	1.94	43.63
proximal part	0	5.0	13.0	13.2	2.52	56.63
*m. gastrocnemius*						
lateral head	17	1.9	4.0	4.4	2.12	47.71
medial head	12	2.7	5.5	5.7	1.98	44.45
*m. Plantaris (FDL)*	21	2.1	10.0	10.2	4.24	95.46
*m. biceps femoris*	26	6.9	25.0	25.7	3.17	71.43
*m. semitendinosus*	16	9.5	13.5	14.5	1.39	31.26
*m. semimembranosus*	16	9.5	8.0	8.7	0.83	18.70
*m. rectus femoris*	20	2.3	8.0	8.2	3.10	69.65
*m. gracilis*	0	5.7	5.0	5.2	0.86	19.29
*m. sartorius*	0	12.0	6.0	6.3	0.50	11.15
*m. caudofemoralis*						
superficial head	40	7.1	4.5	4.7	0.48	10.86
deep head	14	6.8	4.0	4.0	0.54	12.24

**Figure 2:**
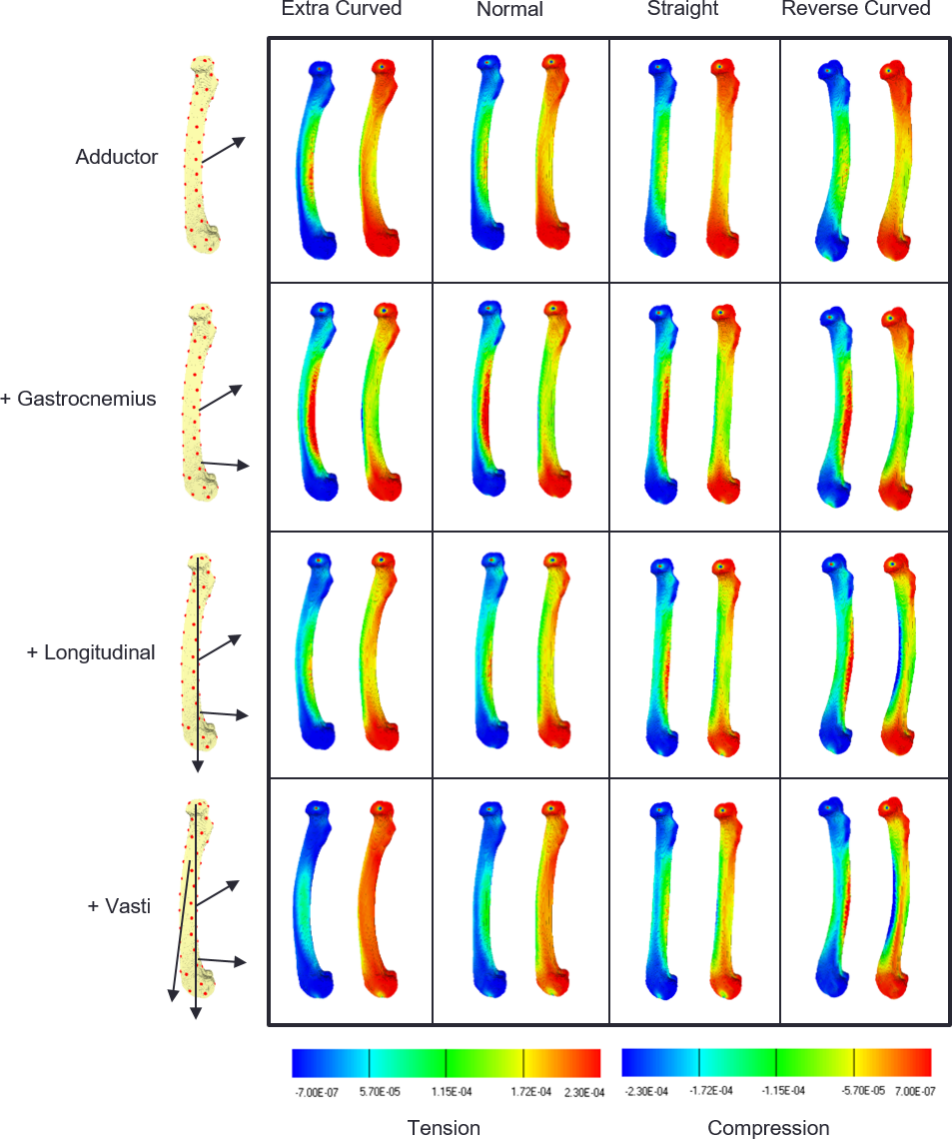
Strain contour maps. Strain maps of the four models under the cumulative load conditions. The first row, adductors only, then gastrocnemius is added, followed by the longitudinal force and then the vasti. Each panel contains a tension map and a compression map. Regions of high tension in the tensile maps are shown in red. Regions of high compression in the compressive maps are shown in blue. Bending is indicated by a colour gradient across the diaphysis.

Strain contour maps for the successive addition of loads on the four models are shown in Fig. 2. The first row shows that the adductor force causes caudal bending in all the models, as shown by the elevated tensile strains on the caudal surface (warmer colours in the left-hand map in each frame), and the elevated compressive strain on the cranial surface (cooler colours in the right-hand map in each frame). The second row shows that the addition of gastrocnemius loads increase this cranial bending in all the models. In the third row, longitudinal loads are applied and these have their greatest effect on the extra-curved and normally curved models, reducing cranial bending. In the last row, the vasti force is added to the other loads and the cranial bending strains are further reduced in the normal and extra-curved models. However, in the straight model the reduction in cranial bending is minimal, and in the reverse-curved model the compression on the cranial side is further increased, indicating that these last two loads add to the level of cranial bending.

**Figure 3:**
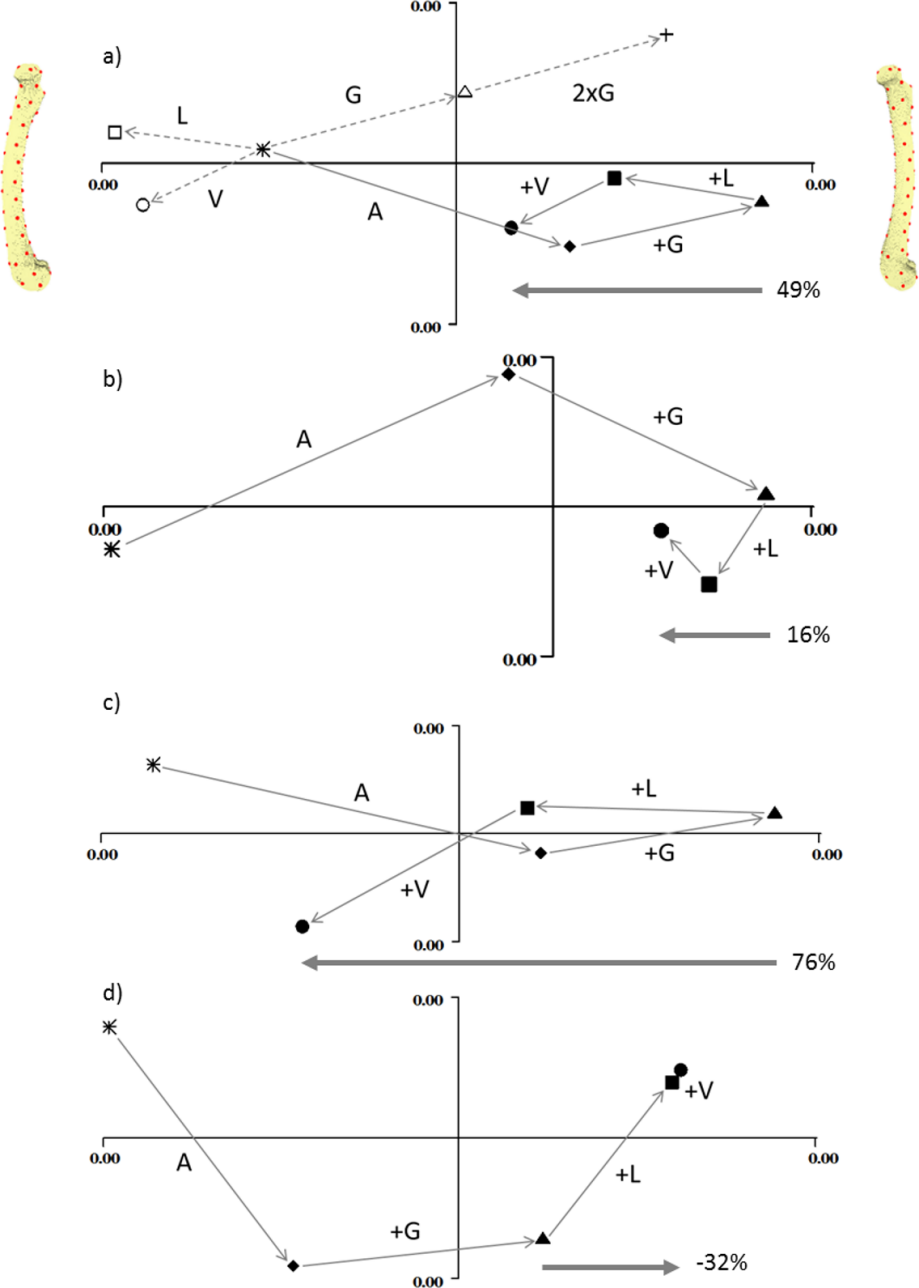
Geometric morphometric analysis of deformation due to loading. Individual principal components plots of the four models under the four load conditions (A) normal, (B) straight, (C) extra-curved and (D) reverse curved. In (A), the open symbols are: circle, vasti only; square, longitudinal only; triangle, gastrocnemius only. The cross in (A) demonstrates that doubling the gastrocnemius force magnitude doubles the vector displacement in shape space. In all figures, the filled symbols are: star, unloaded; diamond, adductor only; triangle, adductor and gastrocnemius; square, adductor, gastrocnemius and longitudinal; circle, adductor; gastrocnemius, longitudinal and vasti. In all figures, PC1 is the *x* axis and PC2 is the *y* axis. Along the *x* axis, caudal bending is to the left and cranial bending is to the right, which is indicated by the models at either end of the *x* axis in (A). These are visualisation aids only, not “end-points”. In each figure the arrow and percentage indicates “the curved bone effect”, the extent to which the longitudinal and vasti forces can counter the cranial bending due the adductors and gastrocnemius.

Figure 3 shows the deformation produced by the addition of each load on each of the four models. In each plot, PC1 (the *x*-axis) represents cranial bending to the right and caudal bending to the left (as indicated by the pictures on either side of the first plot). In each case the unloaded state (star) is on the left, and the adductor (diamond) and gastrocnemius (triangle) loads move the bone into cranial bending. In the normally-curved femur (3a) the longitudinal and vasti loads reduce the cranial bending by about half. In the straight model (3b) the longitudinal and vasti loads reduce the cranial bending by less than 20%. In the extra-curved model (3c) the longitudinal and vasti loads reduce the cranial bending by more than 70%. However, in the reverse-curved model, the addition of longitudinal and vasti loads actually increases the cranial bending.

**Figure 4:**
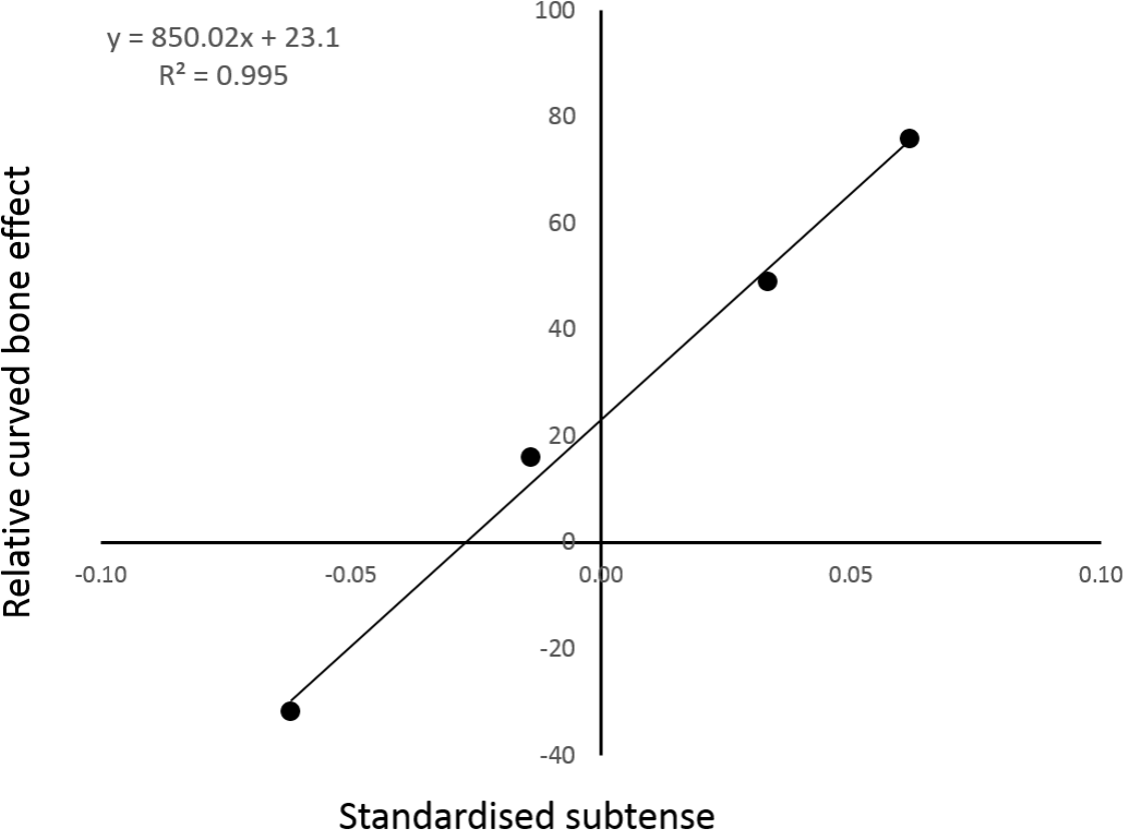
The relative strength of the curved bone effect appears to increase linearly with the degree of caudal curvature. The *x* axis indicates the standardised curvature measurement, with caudal curvature to the right and cranial curvature to the left. The *y* axis is the percentage of cranial bending overcome by the caudal-bending curved bone effect in each of the four individual PC analyses. The points from left to right are: reverse-curved, straight, normal and extra-curved.

There appears to be an almost linear relationship between the model curvature and the percentage of cranial bending mitigated by the combined action of the longitudinal and vasti loads (Fig. 4).

## Discussion

This study has demonstrated that models of caudally-curved quokka femora are less strained than the straight or reverse-curved models. In the caudally curved models the vasti and longitudinal forces generate caudal bending—the curved bone effect—that can counter the cranial bending due to adductor and ankle plantarflexor forces. In the straight model, the curved bone effect is too small to effectively balance the cranial bending strains. In the reverse-curved model the curved bone effect is operating to exacerbate the cranial bending. This study raises a number of issues that are further explored in this discussion. Several hypotheses have been proposed to explain the existence of curvature in long bones, but none account for the direction of curvature. The forces we applied were 10% of the maximum possible forces based on PCSA, but there is no reason to assume that all the muscles operate at the same proportion of their potential, and it seems likely that in reality the vasti and longitudinal forces are relatively larger than the adductor and ankle plantarflexor forces. The degree of curvature of the models seems to bear a linear relationship with the amount of bending induced by the longitudinally acting forces (curved bone effect).

The transverse forces from the adductor and gastrocnemius muscles produce cranial bending strains to a similar degree in all four models (Figs. 2 and 3). There are small differences which result from the fact that, although the size and direction of the forces are constant, the models have different curvature, and so the line of the shaft with respect to the forces and the length of the model are slightly different. However, the longitudinal forces have markedly different effects on the four models. The longitudinal forces have very little effect on the straight model. On the normal and extra-curved models, the longitudinal forces have caudal bending effects that increase with curvature, and on the reverse-curved model the same forces have a cranial bending effect. The overall result is that in the normal and extra-curved models the longitudinal and transverse forces cause bending strains that cancel each other out. Thus, we can say that in the case of the quokka femur, the curvature has a strain-reducing effect, and this supports the ideas of Pauwels (1980, Fig. 12) and Frost (1964, Figs. 5 & 7). This reinforces the theory that bone curvature is an adaptation to reduce bending strain in bones that are subjected to habitual bending loads (Milne, 2016).

Various other hypotheses have been proposed to explain the curvature seen in long bones: that curvature exists to create a strain gradient to stimulate bone remodelling for tissue maintenance (Lanyon, 1980); that the curvature exists to provide a warning of approaching strain limits (Currey, 1984); or that the curvature makes the direction of bending strains predictable (Bertram & Biewener, 1988). These ideas all suffer from the same weakness: they would work equally well regardless of the direction of the curve. However, among terrestrial quadrupeds, the bones all have the same direction of curvature. The present study demonstrated that reversing the direction of curvature has the effect of magnifying the bending strains, which would be neither adaptive nor beneficial. Therefore, it is apparent that the direction of curvature is of biomechanical significance.

### Applied loads and their effect on the results

Our results seem to indicate that the extra-curved model is less strained than the normal model and this raises an obvious question: why isn’t the quokka femur more curved than it is? The forces applied in the modelling performed here were based on 10% of the maximum possible force for each muscle. For the normally-curved model, the gastrocnemius and longitudinal forces would need to be doubled in order to completely neutralise bending due to the transverse forces.

It is very likely that the caudal bending forces used in this analysis were underestimated. In particular, the longitudinal force was very difficult to quantify. In life it would be comprised of components from joint reaction forces, gravity and spanning musculature. In this study only the component from the spanning musculature was accounted for, as it was the only easily-quantifiable component. While this conservative estimate of the longitudinal force was likely the greatest source of error, it is also probable that the vasti force was underestimated. The vasti would be expected to generate enough force to produce an extension moment at the knee, not only to resist the ground reaction force, but also to resist the flexion moment caused by the gastrocnemius and hamstrings.

The actual forces that quokka femora are subject to during stance phase are not known—indeed, no reference was found to any similar data on any marsupials or even other mammals of similar size. PCSA was therefore chosen as a “best estimate” method. Faced with the same problem, Milne (2016) assigned each force vector a magnitude equal to body weight. PCSA preserves the relative force-magnitude of the muscle groups, such that larger groups have a correspondingly larger effect on the model. However, initial trials soon revealed that using 100% PCSA was unviable, as it produced macroscopic deformation in the models. So, the PCSA values were scaled to 10%, which gave force values that were remarkably close to body weight, which lends considerable credibility to Milne’s methodology. This scaling of the muscle forces may appear arbitrary, however, it is of little relevance to the results. Regardless of the absolute magnitudes, if the forces are scaled in proportion to each other—which they were—the results will be the same; the *relative* effects of each of the muscle groups will be preserved.

PCSA-scaled muscle forces assume that all the muscles are active to the same degree at the same time. This, although potentially unrealistic, was unavoidable. However, the PC plots contain unique vector properties that allow other force combinations to be estimated from the existing data. The displacement on the PC plots caused by each muscle group is directly proportional to the magnitude of the force. This means that by simple vector arithmetic, alternative magnitude combinations can be tested without the need for further simulations (O’Higgins & Milne, 2013).

### The mathematical proof

Placing the normal model in neutral bending would require an approximately twofold (2.04) increase in the caudal bending forces, relative to the cranial bending forces. Comparatively, to achieve the same effect in the straight model would require that the caudal bending forces are 6.32 times larger relative to the cranial bending forces. The extra-curved model, on the other hand, would only require that the caudal bending forces be relatively 1.32 times larger. This indicates that the extra-curved model, when fully loaded, is subject to less bending strain than the normal model. However, the manipulations required to place the normal model in neutral bending would place the extra-curved model in caudal bending, which would be consistent with the predictions of this study.

The inclusion of the two extra form-variant (i.e., extra- and reverse-curved) models facilitated analysis of the relationship between sagittal curvature and the curved bone effect. Indeed, regression analysis strongly suggested a linear relationship between the degree of sagittal curvature and the fraction of cranial bending overcome by the curved bone effect. This linear regression analysis contained only four data points, so it would be premature to cite this data as conclusive. There does appear to be a strong trend, although interpolation of further data points would be required to determine the mathematical nature of the relationship present.

Intuitively, the curvature moment arm (subtense) is the mechanical purchase on which a longitudinal load acts. Therefore, the positive relationship found in Fig. 4 is not unexpected. The only remaining question is: is this relationship linear? Swartz (1990) observed that the expected cortical bending stress is directly proportional to the curvature moment arm (subtense). While this strongly suggests a linear relationship between bending moment magnitude and initial curvature, it does not completely describe the observed trend in Fig. 4. However, it is possible to demonstrate mathematically that the observed trend is indeed linear.

**Figure 5:**
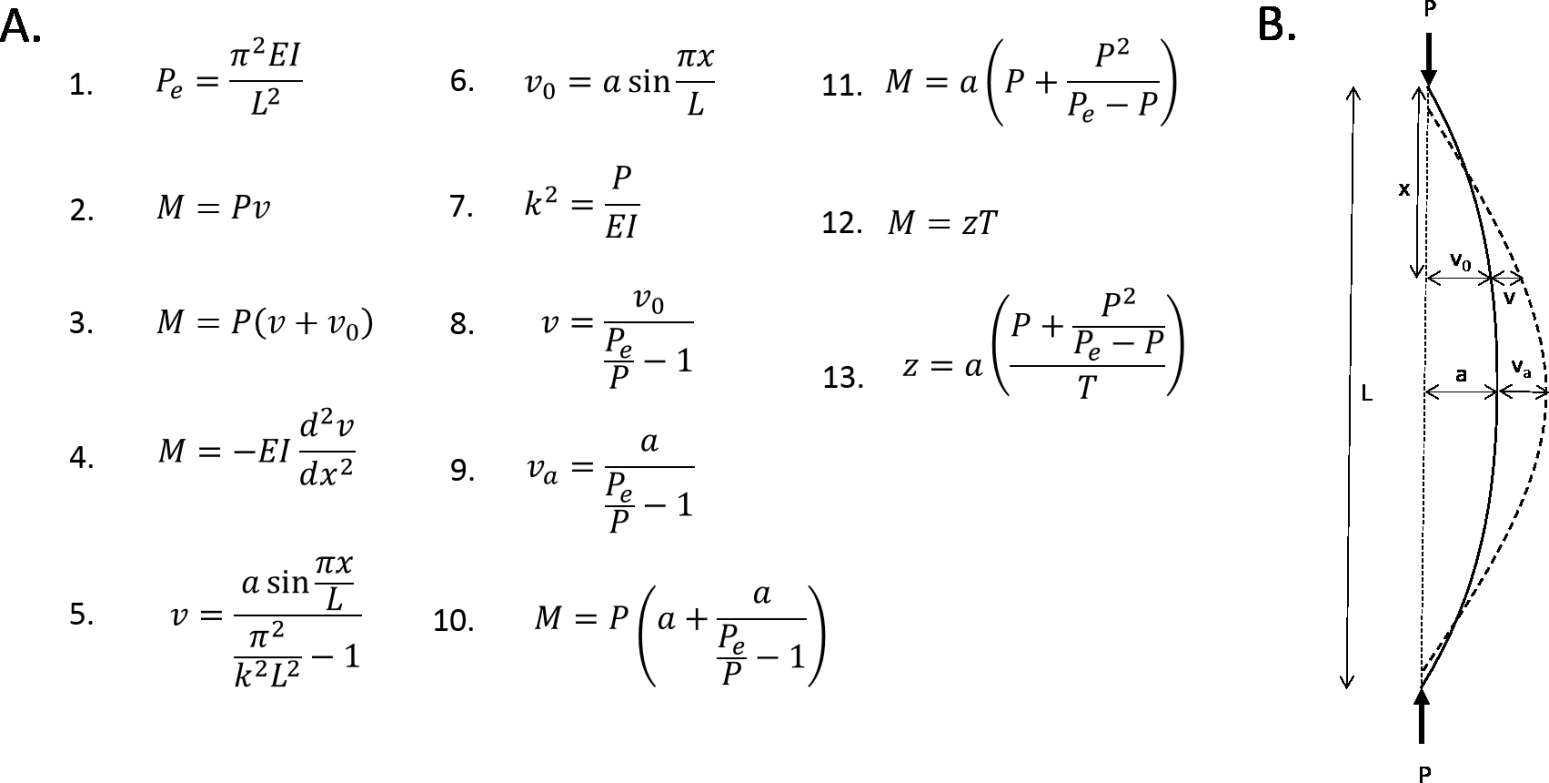
Mathematical formulae explaining the linear relationship between curvature and bending. (A) Formulae referred to in the text. (B) Diagrammatic representation of variables.

The femur can be modelled mathematically as an idealised curved beam with a hollow cylindrical cross section (note that the following analyses are adapted from Case, Chilver & Ross, 1999) (see Fig. 5). All four femoral models are assumed to have the same chord length (*L*), longitudinal load magnitude (*P*), material properties (*E* = Young’s modulus), and diameter (*I* = cross-sectional area moment of inertia). Straight beams, when loaded axially, will buckle under a critical load (Euler load, *P*_*e*_, Eq. 1). At this point, the bending moment at an arbitrary distance (*x*) along the chord is the product of the longitudinal load and the induced curvature (*v*, Eq. 2). If the beam has an initial curvature (*v*_0_), then the bending moment is the product of the longitudinal load and the total curvature at that point (Eq. 3). If the beam is initially unloaded, then the bending moment at an arbitrary distance (*x*) along the chord is proportional to the change in curvature at that point (Eq. 4). Solving this differential equation yields Eq. 5. It is assumed for simplicity that the initial curvature can be modelled by a half a sine curve of amplitude *a* (Eq. 6). The resistance of a beam to bending (*k*) is described in Eq. 7. This equation, as well as Eq. 6 can be substituted into Eq. 5. This yields Eq. 8.

The greatest bending moment will be experienced when the curvature moment arm is maximised. This, by definition, is the subtense. So, substituting the subtense into Eq. 8 gives an expression for the expected increase in curvature at the point of maximum initial curvature (Eq. 9). This expression can be substituted into Eq. 3, to give an expression for the bending moment experienced at the point of maximum curvature (Eq. 10). Therefore, this is the maximum bending moment. This equation can be rearranged to give an expression for the maximum bending moment as a function of the subtense (Eq. 11). The longitudinal load and Euler load are consistent between the four models, which means that the expression that is the coefficient of *a* in Eq. 11 is a constant. Thus, the magnitude of the bending moment induced by longitudinal loading is directly proportional to the initial curvature (subtense).

*M* expresses the bending moment induced by the curved bone effect. *T* can be introduced to express the bending moment caused by the habitual loading; this was effectively constant between the four models. In all four models, *M* was only able to overcome a fraction of *T*; this fraction can be expressed as *z*, according to Eq. 12. Equation 11 can be substituted into Eq. 12, and then rearranged to find an expression for z in terms of *a* (Eq. 13). The coefficient of *a* in Eq. 13 is a constant, thus, it is expected that the relationship between the degree of sagittal curvature and the curved bone effect will be linear (Fig. 4).

## Conclusions

This study further developed the theoretical model of bone curvature as a strain-reducing adaptation to habitual loading (Milne, 2016). Furthermore, we present a method whereby this model can be investigated in myriad circumstances. Here we have demonstrated that a transverse muscular bending challenge (read “habitual load”) has the same bending effect on all form variants of a femur, regardless of the sagittal curvature of the bone. The bending effects of longitudinal forces, however, were very much dependent on longitudinal curvature. We showed that the bending caused by longitudinal loading of curvature is proportional to the magnitude of the existing curvature. This means that both the magnitude and direction of curvature in long bones are of biomechanical significance. While this study was conducted in the context of a quokka femur, we suggest that this phenomenon applies to all terrestrial mammals with caudally-curved femora. This understanding adds to previous work showing that the caudal curvature of the radioulna reduces the strains caused by the habitual action of the triceps muscle in weightbearing. Moreover, it increases our confidence that the same mechanism operates in other curved structures in animal limbs. Thus, we conclude that longitudinal curvature in bones presents a manipulable mechanism through which bone can induce a strain gradient to oppose that which is induced by habitual loading.

##  Supplemental Information

10.7717/peerj.3100/supp-1 Supplemental Information 1 Landmarks, surfaces and models

## Additional Information and Declarations

### Data Availability

The following information was supplied regarding data availability: The raw data has been supplied as a Supplemental Information 1.
